# Application of a Nitric Oxide Sensor in Biomedicine 

**DOI:** 10.3390/bios4010001

**Published:** 2014-02-04

**Authors:** Carlota Saldanha, José Pedro Lopes de Almeida, Ana Santos Silva-Herdade

**Affiliations:** 1 Instituto de Bioquímica, Instituto de Medicina Molecular, Faculdade de Medicina de Lisboa, Edifício Egas Moniz, Av. Prof. Egas Moniz, 1649-028 Lisboa, Portugal; E-Mails: jpedro.gla@gmail.com (J.P.L.A.); anarmsilva@fm.ul.pt (A.S.S.-H.); 2 Servico de Imunoalergologia, Centro Hospitalar Lisboa Norte-Hospital de Santa Maria, 1649-028 Lisboa, Portugal

**Keywords:** endothelium erythrocyte nitric oxide, microelectrode

## Abstract

In the present study, we describe the biochemical properties and effects of nitric oxide (NO) in intact and dysfunctional arterial and venous endothelium. Application of the NO electrochemical sensor *in vivo* and *in vitro* in erythrocytes of healthy subjects and patients with vascular disease are reviewed. The electrochemical NO sensor device applied to human umbilical venous endothelial cells (HUVECs) and the description of others NO types of sensors are also mentioned.

## 1. Introduction

Nitric oxide (NO) is a soluble gas synthesized from L-arginine by the enzyme nitric oxide synthase (NOS) [1]. Three isoforms of NOS have been described, namely the neuronal (nNOS or NOSI), the inducible (NOSII or iNOS), and the constitutive endothelial form (eNOS or NOSIII), which was the first to be discovered [1,2,3]. Both neuronal and endothelial enzymes are activated by the calcium and calmodulin complex, and the inducible form binds to calmodulin but is independent of intracellular calcium concentration [4]. These isoenzymes are flavoproteins that act on L-arginine, in the presence of oxygen and NADPH, and require tetrahydrobiopterin (BH4). In the absence of BH4, a superoxide anion is formed instead of NO [5]. The isoform eNOS is also expressed in platelets and in cardiac myocytes [6,7].

NO produced in the endothelial cell diffuses to the lumen where it is captured by red blood cells (RBC) and transferred into muscle cells where it induces relaxation, eliciting vasodilation. In this mechanism, guanosine 3′,5′-cyclic monophosphate (cGMP), formed from guanosine 5′-triphosphate (GTP) by the action of guanylate cyclase (GC), is activated by NO [3]. cGMP modulates the myosin light chain (MLC) phosphatase positively and MLC kinase negatively, resulting in the dephosphorylation of MLC with subsequent muscle relaxation [3]. NO-induced vasodilation is dependent on hematocrit, blood flow and the hemoglobin free concentration in the circulation [8]. The presence of free hemoglobin reduces vasodilation in pig coronary arteries, induced previously by serotonin or by shear stress [8]. The increase in vascular permeability, inhibition of platelet aggregation, platelet adhesion, proliferation, and migration of smooth muscle cells are effects mediated by NO-dependent cGMP [9].

The intact vascular endothelium establishes a dynamic interface between blood and tissues, allowing gas and metabolites exchanges and participates in hemostasis, in thrombosis, and in inflammatory and anti-inflammatory mechanisms [10,11]. The phenotype of endothelial cells is dependent on the location in the vascular field and presents specific responses to various stimuli [11]. Endothelial cells of post capillary venules respond to inflammatory signals and those in the arterial vascular network release vasoactive substances into the blood [11]. Among the endogenous vasoactive compounds, acetylcholine (ACh) acts like an autocrine or paracrine signal in endothelial cells, stimulating eNOS, with the formation of NO [1,3].

The rapid time course of these events and the short half-life of NO includes several methods, based on electrochemical, chemiluminiscent, and spectrophotometric principles, which have been developed in order to measure NO or its derivative molecules. The method must be sensitive for *in situ* measurements. NO microsensors have been developed for real time assessment *in vivo* [12,13,14]. The real time measurement of NO, in response to stimuli and drugs, will contribute to therapeutic advances in endothelial dysfunction.

## 2. Nitric Oxide Sensors

The utilization of NO sensors allows the quantification of NO concentration ranging from subnamolar to micromolar values [15]. Usually, the biosensor consists of a biorecognition element, a signal transducer and a detector. The electrochemical sensors are, for now, the more reliable tool for NO detection in real time. They operate via the application of a potential at the electrode surface positive or negative to electrochemically oxidize or reduce NO. The resulting transfer of electrons is measured as a current proportional to the NO concentration.

Our studies of NO measurement in human erythrocytes suspensions were performed with the amiNO-IV sensor (Innovative Instruments Inc., Tampa, FL, USA) [16]. The figure of the electrode can be seen in the webpage: http://www.2in.com.

The sensor has a sharp metallic tip completely covered with a series of membrane, including a gas permeable membrane. The “amiNO” series of nitric oxide sensors, with tip diameter ranges of 7 µm to 600 µm, do not require an external reference electrode has high sensitivity abolishing the errors due to baseline drift associated with temperature changes and they are shielded from electrical noise. They were designed for *in vivo* and large surfaces (cultured cells), works with the inNO-T meter with easy calibration procedures. The inNO-Tcombine both a NO configured potentiostat and a software controlled data acquisition system included in one battery powered unit. The “amiNO” series sensor is covered with a triplecoat gas permeable membrane to guarantee selectivity and fast response time. The NO diffuses through the membrane and is then oxidized at the working platinum electrode, resulting in an electric current. The redox current is proportional to the NO concentration outside the membrane and is continuously monitorized with an inNO-TM software (version 1.9 supplied by Innovative Instruments Inc.) installed on a PC computer. The calibration curve and its representative appear in Figure 1 of our published previous work [16]. Briefly, the sensor is calibrated by a simple, economical, and a reliable chemical reaction for NO production. This reaction is based on the conversion of nitrite to nitric oxide in acidic solution in the presence of iodide ion. The reaction has a ratio of one to one, meaning that the amount of NO produced in this reaction equal to the amount of nitrite added.

The amino-IV sensor with its NO-permeable membrane triplecoat avoid a broad range of interfering molecules forming during the electrochemical reaction of NO on metal surfaces at positive electrode potentials via electron oxidation mechanism: Alloys of platinum [17], carbon fibber [18], and glassy carbon [19] are materials additionally developed to cover the surface of the electrodes that show variable sensitivity, selectivity, and signal stability [20,21,22,23].

The most common modes of electrode operate are by electroreduction of NO, direct electrooxidation of NO and catalytic electrooxidation of NO [20,21,22,23]. The electroreduction has the advantage to eliminate the interfering molecules but, at variance, has low sensitivity and pH and electrode surface characteristics dependence [24,25,26]. In these types of electrode oxygen molecules interferes and is a problem in biological applications due to its scavenger properties as mentioned in the previous sections. In the electrode operate by electroreduction of NO the introduction of a transition metal or metalloproteins such as haemoglobin have proven to be useful to improve sensitivity and measurements of NO at low range of sub-micromolar concentrations [24,25].

In the electrode operate by the direct electrooxidation of NO there are broad types of sensors with different electrode material composition of NO-selective membranes, and diverse diameters originating a large variety of values of limit of detection ranging from 0.083 nM to 75 nM [27,28]. Thus, they are dependent on the permselective membrane. In the type of electrode that operates by catalytic electrooxidation of NO a redox mediator for example a metalloporphyrins immobilized on the electrode surface or incorporated in a polymer is utilized [18]. The function of the mediator is to acts as catalysts for the oxidation of NO. However, non-porphyrin complexes have showed also similar results [29].

The sensors descried in the literature to measure NO in solution are classified as belonging to three classes as follows: Shibuki-style; solid permselective; and solid catalytic and their characteristics and composition are summarized in the Table 1 [20,30]. The first one style of sensor determine NO by electrooxidation and the others two by electrooxidation or electroreduction [20,30]. All the three types of sensors integrate a reference electrode that is within the electrolyte filling solution in the Shibuki-style. The catalytic style comprise a mediator (metalloporphyrins or metal phtalocyanines) for catalyze the oxidation or reduction of NO.

While sensors applied to *in vivo*, NO measurements in blood has confined in humans [31] others for determinations of NO released in biological tissues like heart, brain, or lung are used only in animal experimental models [32,33,34]. For example an electrochemical microsensor has been inserted into a human hand vein to detect NO in blood vessels of healthy persons [31]. It is confirmed *in vivo*, in human beings, that the endothelium derived relaxing factor is the NO from the stimulation with ACh [31].

**Table 1:** Characteristics and composition of nitric oxide sensors.

Sensor class	Internal filling solution	Composition	Sensitivity	Miniturization
Shibuki-style	Electrolyte	Platinum and silver	Variable over time and between sensors	Not possible
Solid permselective	Eliminated	Carbon	Multiple membranes discriminate interference molecules	Possible
Solid catalytic	Eliminated	Mediator incorporated in electrode surface or in permselective membrane	Minimize interference molecules	Possible

## 3. Nitric Oxide in Arterial Endothelium

The vasoactive function of ACh could be compromised by the erythrocyte aggregation tendency that is increased in a few vascular disorders including hypercholesterolemia, arterial hypertension, acute myocardial infarction, and diabetes [35,36,37,38].

The vascular endothelium is dysfunctional when it is not able to regulate its tone to maintain structural organization contributing to the installation and progression of hypertension and atherosclerosis [11,39]. These arterial diseases are considered cardiovascular risk factors and are associated with stimulation of NAPH oxidase and generation of reactive oxygen species [40,41]. Dismutation of the superoxide anion hydrogen peroxide is formed, stimulating the expression of eNOS that was verified to be associated with these two cardiovascular risk factors [42,43]. In this case, NO production is insufficient to overcome consumption by superoxide anion with the generation of peroxynitrite that deregulates (uncouples) eNOS, switching to the production of superoxide anion instead of NO [44].

Vasodilation of the vessels fails to appear by lower concentrations of NO, which support platelet and leukocyte adhesion stimulating the inflammatory response in the pathogenesis and progression of atherosclerotic disease [45]. Its etiology is recognized as complicated and multi-factorial [45]. Thus, any manipulations of the pathway of eNOS appear to be promising treatments [46,47].

In the composition of the atheromatous plaque there are muscle cells, macrophages that are in the apoptotic state and are ingested by phagocytes, which decrease the inflammatory response and plaque regression [48]. Pro-apoptotic functions and anti-atherogenic properties of NO are important, along with destabilization of the plaque, which could occur due to enhancement of muscle cell apoptosis induced by NO [48]. The most effective way to increase NO synthesis is by adaptation to hypoxia that is observed in hypertensive stage 1 patients characterized by decreased concentrations of NO [49,50]. Patients with grade 1 hypertension submitted to intermittent conditions of normobaric and hypoxia normalize blood pressure and NO synthesis [50].

The constant presence of hypoxia can lead to decreased synthesis of NO and in turn to the onset of pulmonary arterial hypertension. Inhaled ethyl nitrite has shown promising results in neonates with pulmonary hypertension [51]. Inhibitors of the enzyme phosphodiesterase that catalyze the decomposition of cGMP may be an alternative therapy to the inhalation of NO applied in pulmonary hypertension [52,53]. Nitroglycerin (GTN) acts by inducing vasodilation independent of the endothelium and is used as a therapeutic agent in coronary artery disease with some conditions [54]. Chronic use of NTG regress NO levels and maintains endothelial dysfunction [54].

There are many exogenous compounds leading to nitric oxide availability including nitrite, S-nitrosothiols, N-nitroso-proteins, and iron nitrosyl complexes [55]. Nonsteroidal anti-inflammatory compounds (NSAID) related to derivatives of NO molecules have been synthesized to verify its effectiveness as NO donors and as cardioprotective agents [56]. Among the NSAIDs, aspirin derivatized with NO donors decreases intestinal toxicity and stimulates the eNOS enzyme in a pulsatile manner [56,57]. The statins, the type 1 receptor blockers of angiotensin II and estrogen, increase the synthesis of tetrahydrobiopterin. Statins also inhibit NADPH oxidase and protect the cofactor from oxidation induced by eNOS [58]. Polyphenols present in red wine, by inhibiting the overexpression of NAPH oxidase reduce oxidative stress and protect endothelial cells by maintenance of the basal level of NO, reducing the tendency of peroxinite formation [59].

Patients with depression have diminished levels of the natural substrate of NO, L-arginine; it is estimated that depression can lead to the onset of coronary artery disease (CAD) [60]. However, whether supplementation of L-arginine provides improvement for CAD is unknown [60]. It is known that a diet with high levels of black beans, endive, and spinach can be a source of nitrate that gives nitrites in the salivary-enteric circulation, following a sequence of catalysis reaction involving acidic oxides of nitrogen that lead to NO in the blood and tissues [61]. There is evidence that a diet rich in vegetables and supplements of nitrates decreases blood pressure and the risk of brain ischemic occurrences [62]. The risk of cardiovascular events in CAD is associated inversely with the catalytic activity of erythrocyte glutathione peroxidase [63]. In addition, published studies demonstrated the existence of NOS3 polymorphisms associated with some groups of patients with CAD and with groups of insulin-dependent diabetics who will develop CAD [64,65]. The implication of polymorphisms of NOS isoenzymes in the appearance of CAD requires further study.

Recently, measurements of NO *in vivo* and in humans directly using an electrochemical electrode sensor have been developed and applied in the coronary circulation [14]. The study was performed in patients with dilated cardiomyophaty and in healthy controls and NO was measured in the proximal great cardiac vein with a catheter-type sensor [14]. The authors give a contribute for the clinical quantification of endothelial dys(function) by measuring *in vivo* endothelium availability of NO [14]. The determination of NO associated with the evaluation of coronary diameter and coronary blood flow could give useful information for the application of adjusted therapeutics.

## 4. Nitric Oxide in the Venous Endothelium

Human umbilical vein endothelial cells (HUVECs) in the presence of ACh, the natural substrate of acetylcholinesterase (AChE), liberate higher values of NO than basal values [66]. Variance when velnacrine (AChE inhibitor) was added to the HUVECS lowers values of NO than those observed for ACh [66]. These results were obtained using the electrochemical sensor amino-IV NO [66]. Further studies are needed to clarify the signal transduction mechanisms in endothelial cells under the influence of activators and inhibitors of acetylchonisterase. Others biosensors for NO evaluation are available as described above.

The appearance of structural changes in connective tissue, smooth muscle, and of functional changes in venous endothelium, defects in the microcirculatory network, and deficient supplies of nutrients in the venous sector are inducers of varicosity appearance [67,68]. In venous disease, the upright position originates a decrease of oxygen partial pressure in tissues, capillary stasis, and hypoxia that activates endothelial cells with increased cytoplasmic calcium. This is crucial for the release of proinflammatory factors (such as PAF, leukotriene B4, prostaglandins E2 and D2) [69,70,71,72]. The release of histamine and serotonin stimulate the migration of leukocytes to the endothelium and expression of adhesion molecules in both cells [73]. Moreover, endothelial cells produce cytokines (IL-1beta, IL-6, TNF alpha) and prothrombotic factors (von Willebrand factor) that elicit monocytes and activated T lymphocytes with the inflammatory response [73]. Inflammation does not occur only in the post-capillary venules, but also in the large veins which contributes to a better understanding of the etiology of venous thrombosis and pulmonary embolism [74]. Flow-mediated vasodilation is impaired in patients with spontaneous venous thromboembolism, which is an indicator of endothelial dysfunction [75].

In the venous endothelium of healthy individuals, as happens on the arterial side, there is release of nitric oxide which regulates and maintains venous tone [76]. The NO secretion by vascular endothelial cells is depending on shear stress [77]. In chronic venous insufficiency, the diameter of the vein is increased, decreasing shear stress and the production of NO by endothelial cells during the initial phase. After the expression of inducible nitric oxide synthase, higher amounts of NO are released that interact with the superoxide anion (produced by leukocytes and macrophages) producing peroxynitrite that causes tissue oxidation of chronic venous ulcers.

The restoration of basal levels of NO is a therapeutic target for the healing of ulcers [76]. The success of wound healing in patients receiving monocromatic infrared energy and submitted to stretching and resistance exercise may be associated with increases of NO in the blood [78].

If heart failure is accompanied with venous vascular dysfunction, it benefits from therapeutics that acts on restoring venous vascular function dependent on NO [76].

Application of an eNOS inhibitor on the venous network normalizes venous vascular resistance and blood pressure values in the lower limbs, although these results are still preliminary [79].

Hyperemia observed in the elderly with passive movement of the legs is absent in patients with peripheral arterial disease. This supports the hypothesis that NO is present in venous endothelial cells [80]. The results obtained with inhibitors of eNOS confirm this hypothesis [81]. The authors consider that the passive movement of the legs may become a noninvasive instrument for analyzing the function of the endothelial NO in venous tissue [80].

Endothelial function can be evaluated using non-invasive techniques including high-resolution ultrasonography that measures vasodilation in the radial, femoral, and brachial arteries [81]. The vasodilation resulting from the action of vasodilators is used as a benchmark. Nitroglycerin is used as a diagnostic test for portal hypertension (PHT) [82]. The extent of PHT is quantified in clinical practice by measuring the hepatic portal vein pressure gradient [83]. In PHT, the insufficient release of NO from endothelial cells influences the increase of vascular resistance at the level of the intra-hepatic microcirculation [84,85]. There is excessive production of NO in the splanchnic circulation that must be accounted for when NO donors are used to reduce portal pressure [86]. Many of the studies regarding the influence of NO donors on the venous vascular sector are performed in experimental animal models, suggesting the best treatment strategies dependent on NO can function in the resolution of human venous disease.

## 5. Nitric Oxide in Erythrocytes

In physiological conditions, the erythrocyte senses oxygen partial pressure (PaO2) in the vasculature scavenging both oxygen and nitric oxide in higher PaO2 or delivering them in lower PaO2 (NO) [87]. The capture and donation of both gases is dependent on the haemoglobin (Hb) conformation states being its relaxed state is associate with scavenging and its tense state with both gases donation [88,89].

Erythrocytes mediate the availability of NO and collection to and from the endothelium [87]. This recruiting cycle, reservation and donation of NO by erythrocyte applies to both NO synthesized in the lung or inhaled [87].

Previous studies have demonstrated the presence of NO inside the erythrocyte using fluorescence microscopy [90].The influx of NO to red blood cells occurs through the band 3 protein also known by the chloride/bicarbonate anionic channel [91]. The influx depends on the tendency of the band 3 protein to adopt the structure of the dimer or the tetramer and on the degree of denaturation of hemoglobin, *i.e*., the presence of Heinz bodies and methemoglobin [92]. The presence of tetramers or methemoglobin and Heinz bodies are unfavourable and blocks the entry of NO into erythrocytes [12]. The rate of influx in the erythrocytes is higher in the hemoglobin deoxygenated state than in the oxygenated state [93]. Conditions of increased hematocrit favor the influx in oxygenated RBCs [93].

The erythrocyte acts as a carrier and as a nitric oxide donor. The output of NO from the erythrocytes occurs through the band 3 protein dependent on its degree of phosphorylation [94]. The molecular forms of NO, namely the nitrosilhemoglobin (NO bound to heme iron of hemoglobin) the nitrosohemoglobin (NO bind to the thiol group of cysteine 93 of the beta chain of hemoglobin) and nitrosoglutathione regulate the availability of NO by erythrocyte [95].

Glutathione is an abundant molecule inside erythrocytes which has a thiol group that can react with nitric oxide, forming nitrosothiols such as S-nitrosoglutathione (GSNO) [96]. The NO reservoir attributed to glutathione could be influenced by the inactivation of glutathione reductase induced by oxidative stress [97]. The thiol/disulfide reagents such as reduced or oxidized gluthathione (which are present at high levels inside RBCs), have a suitable redox potential that is useful for protein regeneration. For instance, dithiothreitol (DTT) is a thiol-reducing agent capable of regenerating disulfide-containing proteins and also able to establish interchangeable thiol-disulfide reactions with glutathione [98]. The presence of DTT induces erythrocyte changes in enzymatic activity states for example in protein tyrosine phosphatase (PTP) and protein tyrosine kinase (PTK) [99,100]. DTT significantly mobilizes erythrocyte NO to generate nitrites/nitrates and SNOHb, thereby decreasing NO efflux [100]. Using the same experimental model, DTT was proven to enhance the levels of GSNO, nitrite/nitrate concentrations [101].

When auto-oxidation of hemoglobin occurs, it produces peroxide anion which generates peroxynitrite after reacting with NO [102]. The decomposition of peroxynitrite molecules yields nitrite and nitrate [103] and the reaction between peroxynitrite and hemoglobin generates SNOHb, which could decompose to nitrosothiol and nitrate [104]. However, NO may reduce oxyhemoglobin to methemoglobin along with the formation of nitrate without any variation in the methemoglobin concentration [104,105]. This could be associated with the presence of hemoglobin reductase coupled with NADH that is produced in the glycolytic pathway [106]. *In vitro*, the NO donor, Spermine NONOate, induces an increase in the methemoglobin concentration and decreases the P50 values, which means that hemoglobin oxygen affinity increases [105].

An electrochemical method was described for quantification of the efflux of NO from erythrocytes using an NO electrode sensor in erythrocyte suspensions containing acetylcholine [16]. Further studies documented a signal transduction mechanism in erythrocytes involving the AChE-acetylcholine (active enzyme—substrate complex), the Gi protein, the band 3 protein—dependent of the degree of phosphorylation [107,108]. In the presence of velnacrine, an inactive enzyme complex is formed and lower levels of erythrocyte efflux were observed than those obtained in the presence of acetylcholine [107,108]. Timolol is an inhibitor of erythrocyte AChE [109] and does not change erythrocyte NO bioavailability in erythrocyte suspensions [110]. This can be considered an advantage if the vascular lumen is under high levels of reactive oxygen species, as the formation of peroxinitrite will not be favored. The antioxidant properties of timolol have been reported *in vivo* and *in vitro* [111,112,113].

The erythrocyte scavenging property of NO is preserved by fibrinogen binding to the RBC membrane [114]. The same was verified mimicking hiperfibrinogenemia conditions in the absence or presence of acetylcholine [115,116]. However, a reverse situation is obtained when higher fibrinogen concentrations are simultaneously present with phosphorylation of band 3 protein [115]. This result could be considered a useful therapeutic tool in blood storage for further transfusion. Fibrinogen binds to the erythrocyte membrane CD47 and when the agonist peptide of CD47 is added to erythrocytes, the same NO scavenging property was verified [117].

High NO release from RBC samples was observed *in vitro* from patients with hypoxia and inflammatory states, namely sickle cell disease, hypercholesterolemic, and hypertensive patients; also, impairment in erythrocyte deformability was documented [118].

Vasoconstriction and ischemia may occur when patients are submitted to blood transfusions originating from blood-bank-stored blood, which have a lower ability to release both oxygen and NO [119]. NO consumption by erythrocytes is regulated under hypoxic conditions by deoxygenated Hb that binds to iron heme (NO occupies the vacant site left by oxygen). The NO-heme hemoglobin adduct (HbFe (II) NO) has been detected during NO inhalation therapy used for pulmonary hypertension treatment, but it also occurs when deoxygenated blood enters a vascular bed in which NO is produced, such as the pulmonary circulation [120,121]. In situations of hyperemia, the nitrosylated hemoglobin recently measured *in vivo* by a modified subtraction method using electron paramagnetic resonance correlated with the endothelium function measured by tonometry [122]. Under hypoxic conditions established *in vitro* in segments of mesentery arteries of Wistar rats perfused with erythrocyte suspensions vasodilation occurs due to the liberation of NO from erythrocyte in dependence of the shear stress [123].

Inducible NOS expression in the endothelium increases in ischemia/hypoxia conditions or ischemia/reperfusion in which blood flow decreases, thereby favoring HbFe (II) NO formation [124]. From all these results, the use of NO donors must be accompanied by NO *in situ* monitoring with a microelectrode sensor.

## 6. Conclusions

Nitric oxide is a signaling molecule influential in several vascular diseases. By NOS uncoupling, NO lowers its levels and endothelial dysfunction is installed. The endothelial dysfunction generates reactive oxygen species that impair the NO concentration by its combination with superoxide anion. The tendency to apply NO donors seems to be the choice for monitoring the therapeutic option. However, care must be taken with NO measurements *in situ* resulting in the expression of the inducible NOS. The availability of erythrocyte to scavenge or release NO, measured using a microelectrode sensor, is a reflex also of the endothelium and recommended to monitor cardiovascular disease. The signal transduction mechanisms evidenced for the ACh–AChE active complex can be a routed for therapeutic control of NO bioavailability of the erythrocyte. The electrochemical sensors are well established class of *in vivo* sensors, which offer almost real-time NO determinations
